# A Rare Case of Didanosine-Induced Mid-Peripheral Chorioretinal Atrophy Identified Incidentally 11 Years after the Drug Cessation

**DOI:** 10.3390/medicina58060735

**Published:** 2022-05-30

**Authors:** Heba Joharjy, Pierre-Jean Pisella, Isabelle Audo, Marie Laure Le-Lez

**Affiliations:** 1 Department of Ophthalmology, Centre Hospitalier Regional Universitaire de Tours, Bretonneau Hospital, 37000 Tours, France; pj.pisella@chu-tours.fr (P.-J.P.); ml.lelez@chu-tours.fr (M.L.L.-L.); 2 Department of Ophthalmology, King Abdulaziz University Hospital, Jeddah 21589, Saudi Arabia; 3 Unité d’électrophysiologie, Centre Hospitalier National des Quinze-Vingts, 75012 Paris, France; isabelle.audo@inserm.fr; 4 Centre Hospitalier National d’Ophtalmologie des Quinze-Vingts, Centre de Référence Maladies Rares REFERET and INSERM-DHOS CIC 1423, 75012 Paris, France; 5 Institut de la Vision, Sorbonne Université, INSERM, CNRS, 75012 Paris, France

**Keywords:** didanosine, retinopathy, widefield fundus autofluorescence, widefield fundus photography

## Abstract

*Objective:* This article aims to describe a unique case of didanosine-induced retinal degeneration that was discovered 11 years after the drug withdrawal. *Case report:* The patient is a 42-year-old woman with a medical history of HIV and hepatitis C virus since 2004. She has been prescribed antiretroviral therapy since then. For the first seven years (2004–2011), the patient was prescribed a combination therapy consisting of didanosine, efavirenz, and lamivudine. The protocol was changed to atripla (efavirenz, emtricitabine, and tenofovir) from 2011 to 2021. Recently (October 2021–January 2021), the patient was prescribed eviplera (rilpivirin, emtricitabine, and tenofovir). In addition, her past medical history revealed Gougerot-Sjogren syndrome and rheumatoid arthritis. She was prescribed hydroxychloroquine (HCQ) (2009–2021) at a dose of 400 mg daily. She had no vision complaint. *Results:* During her routine HCQ screening at the eye clinic, University Hospital Bretonneau, Tours, France, the widefield colour fundus photograph showed well-defined symmetric mid-peripheral areas of chorioretinal atrophy sparing the posterior pole of both eyes. Furthermore, the widefield fundus autofluorescence illustrated mid-peripheral round well-demarcation hypoautofluorescent areas of chorioretinal atrophy of both eyes. Conversely, the macular optical coherence tomography (OCT) was normal. Many of her drugs are known to be associated with retinopathy such as HCQ, tenofovir, efavirenz, and didanosine. Because our data corroborate peripheral retinal damage rather than posterior pole damage, this case report is compatible with didanosine-induced retinopathy rather than HCQ, efavirenz, or tenofovir retinal toxicity. *Conclusions:* All HIV patients who are presently or were previously on didanosine therapy should have their fundus examined utilising widefield fundus autofluorescence and photography.

## 1. Introduction

According to the World Health Organization (WHO), the global prevalence of human immunodeficiency virus (HIV) infection is estimated to be 0.7 percent [1]. At the end of 2020, 37.7 million individuals worldwide were living with HIV [2]. Every day, 15,000 to 20,000 new cases are reported [3]. Most HIV patients require lengthy treatment with a combination of various drugs, which are known to be associated with retinopathy, such nucleoside reverse transcriptase inhibitors (NRTI) including didanosine, tenofovir, and zidovudine, non-nucleoside reverse transcriptase inhibitors (NNRTI) including efavirenz, and protease inhibitors such as ritonavir [4,5,6,7,8].

Didanosine is a synthetic purine nucleoside analogue belonging to the NRTI category of medicines. It has been licensed by the US Food and Drug Administration (FDA) for the combination therapy of HIV since October 1991. Didanosine toxicity is usually accompanied with mid-peripheral retinopathy, which might worsen long after treatment is stopped [5]. As far as we know, only 26 cases of didanosine retinopathy were recorded between 1992 and June 2021 [6].

We present a case of didanosine-associated mid-peripheral retinopathy that was identified incidentally in an HIV patient who was prescribed the drug for seven years (2004–2011).

## 2. Case Report

### 2.1. Patient Information

A 42-year-old woman was referred to our clinic for hydroxychloroquine (HCQ) maculopathy screening (Table 1 shows the demographic, medical, and drug history information). She had a medical history of HIV and hepatitis C virus since 2004. She has been prescribed antiretroviral therapy since then. The patient was prescribed a combination therapy consisting of didanosine, efavirenz, and lamivudine (2004–2011). The protocol was changed to atripla (efavirenz, emtricitabine, and tenofovir) from 2011 to 2021. Recently (October 2021–January 2021) the patient was prescribed eviplera (rilpivirin, emtricitabine, and tenofovir) (Table 2). In addition, her past medical history revealed Gougerot-Sjogren syndrome and rheumatoid arthritis since 1995. She had been prescribed hydroxychloroquine (HCQ) since 2009 at a dose of 400 mg per day. Her cumulative dose of HCQ was 1800 g.

She was referred to the eye clinic, University Hospital Bretonneau, Tours, France, for routine HCQ retinal toxicity screening. The patient reported no visual symptoms. Her last CD4+ count was 416 copies/mL, and her HIV plasma viral load was undetectable (below 20 copies/mL). The patient neither had a personal history of cytomegalovirus (CMV) retinitis or HIV retinopathy nor a family history of eye disease.

### 2.2. Visual Examination

**Figure 1:**
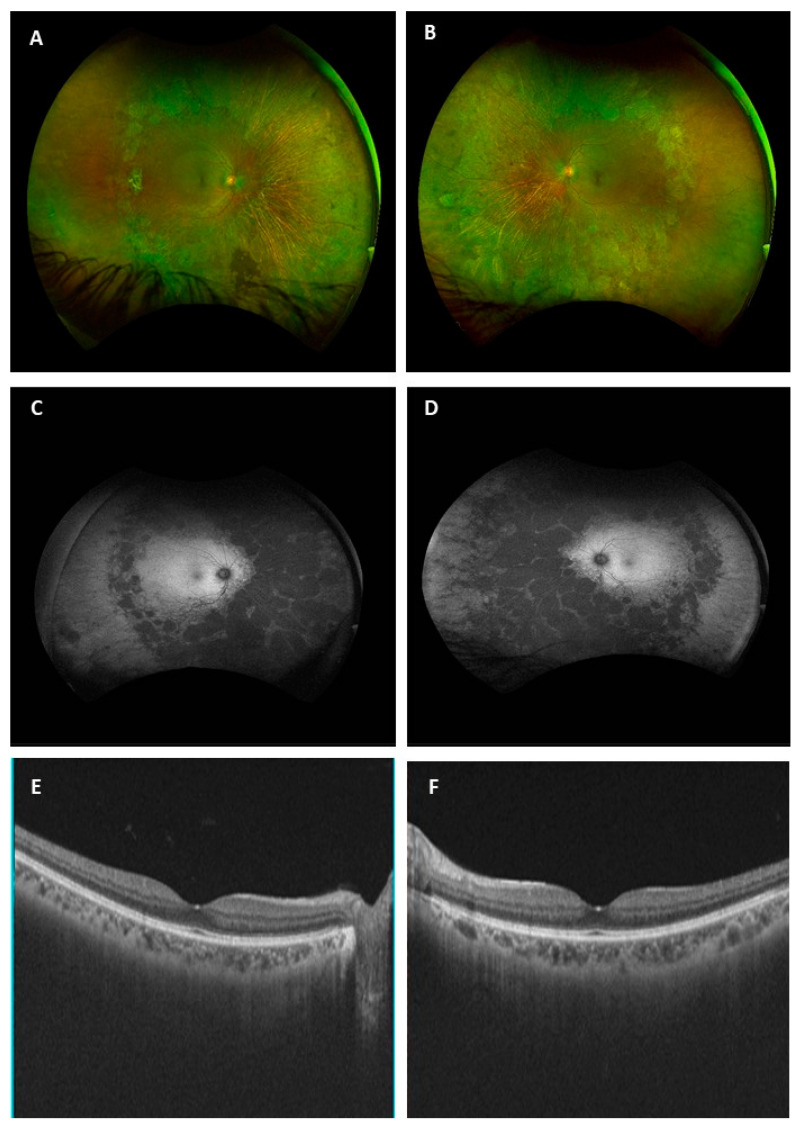
Ultra-widefield fundus photographs of the right (**A**) and left (**B**) eye performed using the optos camera showed well-delineated concentrical mid peripheral patches of chorioretinal atrophy sparing the macula in both eyes. Ultra-widefield short-wavelength fundus autofluorescence illustrated mid-peripheral round well-demarcated patchy loss of autofluorescence in both eyes (**C**,**D**). Macular Optical coherence tomography (OCT) photos showed no macular abnormality in either eye (**E**,**F**).

**Figure 2:**
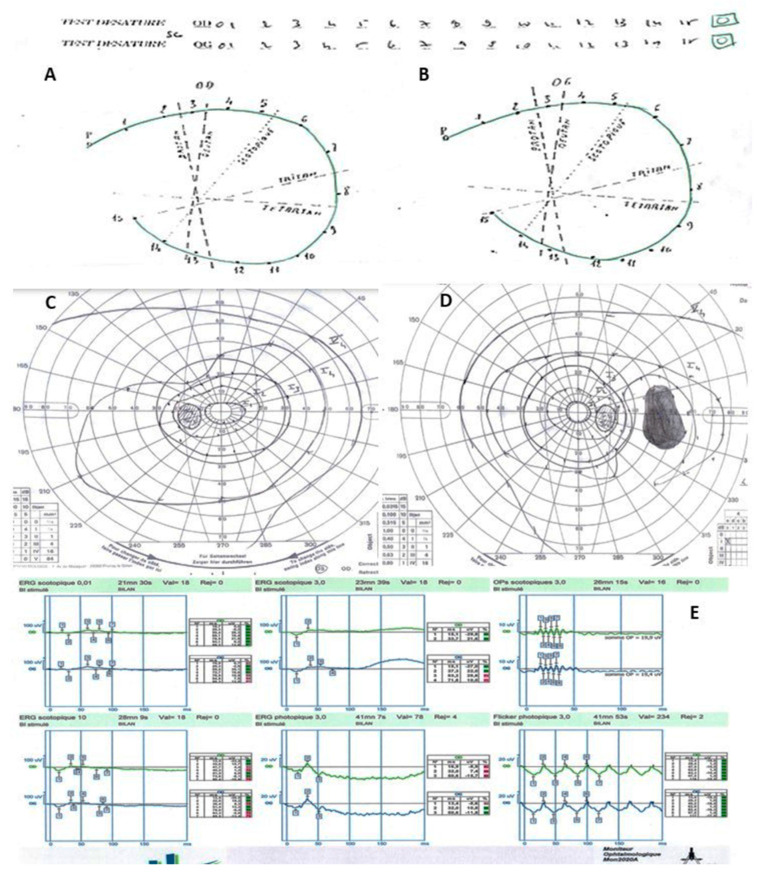
Farnsworth D-15 colour vision tests showed no abnormality in either eye (**A**,**B**). Goldmann kinetic visual field revealed (**C**) a superior temporal depression with the I4e isopter in the left eye and (**D**) absolute scotomas in the temporal mid-peripheral field between 30° and 60° in the right eye. A full-field electroretinogram (ERG) revealed moderate generalized rod and cone dysfunction in both eyes (**E**).

**Figure 3:**
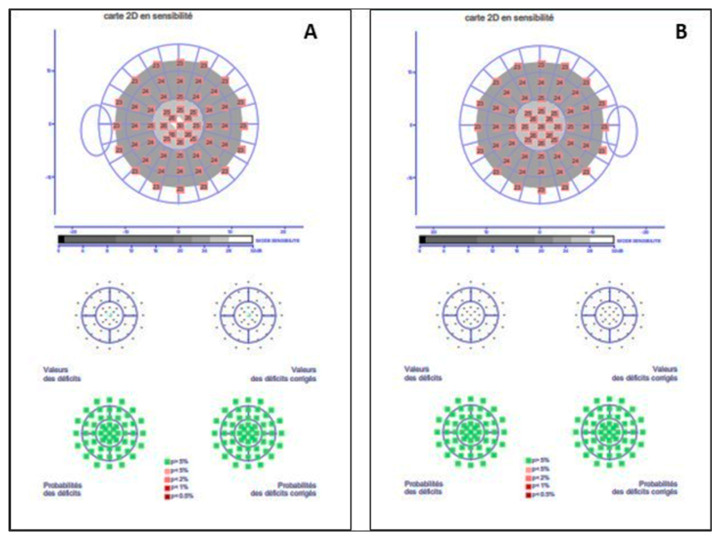
Static visual field test within the 12 central degree FAST 12 perimetry Metrovision visual field test showed no central field defects in either eye (**A**: left eye; **B**: right eye).

On biomicroscopic examination, the best-corrected visual acuity measured using a logMAR chart (Logarithm of the Minimum Angle of Resolution) was 0.00 logMAR in both eyes. The intraocular pressure was 11 mmHg in both eyes. The slit-lamp examination was unremarkable in both eyes. The widefield colour fundus photograph (OPTOS California Ultra-widefield non-mydriatic fundus camera) showed well-defined symmetric mid-peripheral areas of chorioretinal atrophy sparing the posterior pole of both eyes (Figure 1A,B). The widefield fundus autofluorescence (OPTOS California Ultra-widefield non-mydriatic fundus camera) illustrated mid-peripheral round well-demarcation hypoautofluorescent areas in the peripheral retina of both eyes (Figure 1C,D). Macular optical coherence tomography (TOPCON DRI Swept Source OCT) of the posterior pole was normal for both eyes (Figure 1E,F). The colour vision test (Farnsworth desaturated 15 Hue colour test) was unremarkable (Figure 2A,B). The Goldmann kinetic perimetry visual field showed a superior temporal depression with the I4e isopter in the left eye and absolute scotomas in the temporal mid-peripheral field between 30° and 60° in the right eye (Figure 2C,D). A full-field electroretinogram (ffERG, MonPackONE, METROVISION), conducted according to the International Society for the Clinical Electrophysiology of Vision (ISCEV) standards, revealed moderately generalized rod and cone dysfunction in both eyes (Figure 2E). The static semi-automated visual field with the 12 central degree was unremarkable (Figure 3).

## 3. Discussion

Because of the advancement of antiretroviral medications, the majority of HIV patients now enjoy long-term remission. Often, a cocktail of several drugs is required to stabilize the disease’s progress. Unfortunately, unwanted side effects might arise with long-term therapy, and determining which medicine is the cause can be difficult [6]. While HIV retinopathy, opportunistic infections, and some malignancies are the most common causes of retinal problems, toxic medication effects must also be mentioned. HIV patients are frequently subjected to many drugs, including clofazimine, zidovudine, didanosine, efavirenz, and ritonavir, all of which have been linked to retinopathies [7,8].

Here, we report a case of peripheral retinopathy that was incidentally diagnosed during regular HCQ screening at our eye clinic, University Hospital Bretonneau, Tours, France. This case involved a woman with a history of HIV, hepatitis C virus, Gougerot-Sjogren syndrome, and rheumatoid arthritis. By examining the patient’s medication history, we discovered four medications that could cause retinopathy: HCQ, tenofovir, efavirenz, and didanosine.

HCQ is an immunomodulator being used to treat malaria and autoimmune illnesses such as systemic lupus erythematosus and inflammatory arthritis [9]. Treatment with a high dose of HCQ, on the other hand, may result in toxic retinopathy [10]. Perifoveal rods and cones are classically involved in HCQ retinopathy with an initial foveal conesparing [11,12]. Efavirenz, a first-generation NNRTI, is a component of primary antiretroviral therapy [13]. One publication reported few cases of maculopathy secondary to efavirenz treatment. Despite the fact of its toxic effect on the retinal pigment epithelium, efavirenz retinopathy usually affects the parafoveal region [14]. Tenofovir, a NRTI, is another antiretroviral drug. Long-term tenofovir use has been linked to retinal pigment epitheliopathy, according to Mehrotra et al. [15]. In the absence of macular involvement, HCQ, efavirenz, or tenofovir retinal toxicity could be excluded.

Didanosine is a synthetic purine nucleoside analogue that belongs to the NRTI group of medicines. It has been licensed by the FDA since October 1991 for the combination treatment of HIV [16]. Didanosine-induced retinal toxicity was first recorded in 1992 with a report of three immunocompromised children treated with didanosine who developed symmetrical bilateral peripheral retinal atrophy that spared the macula. Numerous case reports have documented a characteristic pattern of didanosine-induced retinopathy in HIV-infected individuals, including symmetrical bilateral mid-peripheral concentric retinal atrophy anterior to the posterior pole [5,6,17,18,19,20]. Whitcup et al. demonstrated the absence of macular damage in a child taking didanosine with a histological report of a normal structure of the macular, choriocapillaris, and retinal pigment epithelium [21]. Because our data corroborate peripheral retinal damage rather than posterior pole damage, this case report is compatible with didanosine-induced retinopathy rather than HCQ, efavirenz, or tenofovir retinal toxicity.

Didanosine-related retinal toxicity is usually asymptomatic, and patients retain adequate vision [6]; nevertheless, in advanced cases, the visual field may be significantly compromised [18]. The patient in our situation had undergone treatment with didanosine for seven years (2004–2011) and then stopped 11 years ago; yet, the pathological picture is perfectly compatible with the drug’s toxicity. Furthermore, the majority of the newly identified retinal impairment cases were reported in individuals who had stopped taking didanosine. Given this, it is not unexpected that many patients continue to deteriorate even after stopping didanosine [5]. Despite the discontinuation of the drug, the explanation for the progression of didanosine-induced retinopathy is unknown. One possibility is that the retinal cells were triggered by an irreversible cell death process before treatment was discontinued and, thus, continued to degenerate even after the drug was stopped. The possibility of further toxicity from drugs used as therapeutic switches cannot be ruled out. Other NRTI, such as zidovudine, entecavir, lamivudine, and a protease inhibitor, ritonavir, have been linked to retinopathy. To address this concern, continuous retinal screening of patients taking the new generation of NRTI can be proposed [22].

Because the defects are limited to the mid-peripheral retina, and symptoms are delayed due to macular sparing, diagnosing didanosine toxicity can be difficult. As a result, a thorough fundus examination using multimodal imaging, such as widefield fundus autofluorescence and widefield fundus photography, is beneficial in detecting peripheral abnormalities and monitoring patients [19].

## 4. Conclusions

A case of didanosine-induced mid-peripheral chorioretinal degeneration in an HIV patient with no visual complaints was reported. The case was detected eleven years after the didanosine cessation. All HIV patients who are presently or previously on didanosine therapy should have their fundus examined utilising widefield fundus autofluorescence and widefield fundus photography.

## Figures and Tables

**Table 1:** The patient demographic, medical, and drug history information.

	Patient Characteristic
Age	42 years
Sex	Female
Ethnicity	Non-Hispanic
Race	African
Weight (kg)	51 kg
Height (cm)	167 cm
BMI (kg/m^2^)	18.3
Marital status	Married
Tobacco use	3–4 cigarettes per day since 2003
Alcohol use	No
Past medical history	▪ HIV since 2004 ▪ Hepatitis C since 2004 ▪ Gougerot-Sjogren syndrome since 1995 ▪ Rheumatoid arthritis since 1995
Past surgical history	Appendectomy
Allergy status	No known drug allergies
Family history of eye diseases	No known family history
Drug history	▪ Hydroxychloroquine (400 mg/day, cumulative dose 1800 g, 7.5 mg/kg) since 2009 ▪ Prednisone (5 mg/day) since 2010 ▪ Calcium/vitamin D3 (500/440 IU) since 2010 ▪ Esomeprazole (20 mg/day)

**Table 2:** Antiretroviral treatment taken by the patient from 2004 to January 2022.

Treatment Start	Treatment End	Antiretroviral Therapy
2004	November 2011	Combination of 250 mg didanosine, 600 mg efavirenz, and 300 mg lamivudine
December 2011	October 2021	Atripla (600 mg efavirenz/200 mg emtricitabine/300 mg tenofovir)
November 2021	January 2022	Eviplera (25 mg rilpivirin/200 mg emtricitabine/245 mg tenofovir)

## Data Availability

Not applicable.
